# The Introduction of Pyrrolotetrathiafulvalene into Conjugated Architectures: Synthesis and Electronic Properties

**DOI:** 10.1002/marc.200800154

**Published:** 2008-05-27

**Authors:** Alexander L Kanibolotsky, John C Forgie, Sergey Gordeyev, Filipe Vilela, Peter J Skabara, Jan E Lohr, Bo M Petersen, Jan O Jeppesen

**Affiliations:** 1 WestCHEM, Department of Pure and Applied Chemistry, University of StrathclydeGlasgow G1 1XL, UK; 2 School of Chemistry, University of ManchesterManchester, M13 9PL, UK; 3 Department of Physics and Chemistry, University of Southern DenmarkOdense M, DK5230, Denmark

## Abstract

A series of new conjugated copolymers incorporating the redox-active pyrrolo-TTF unit has been synthesised. The properties of the polymers have been investigated by cyclic voltammetry and electronic absorption spectroscopy, revealing that the pyrrolo-TTF behaves very differently to its thieno-TTF variant. In comparison to thieno analogues, the band gaps of the new polymers are wider than expected due to a decrease in the polarizability of the heteratom (nitrogen vs. sulfur) and steric interactions between repeat units. Whilst the pyrrolo-TTF units are stronger electron donors than thieno-TTFs in related structures, the two redox active elements of the new polymers (TTF and conjugated chain) function independently under oxidative conditions.

**Figure:**
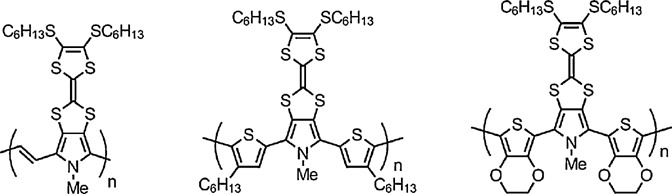

## Introduction

The incorporation of additional redox active components into conjugated polymers (CPs) is an attractive goal for several reasons. The manipulation of the parent polymer’s HOMO and/or LUMO energy levels is one key advantage for device applications, but one can also induce higher stability towards p- and n-doping processes and achieve higher, multi-step redox states. Several well-studied molecular redox systems have been attached to CPs and these include ferrocene,^1^ viologens,^2^, ^3^ fullerenes^4^, ^5^ and tetrathiafulvalene (TTF).^6^–^8^ In the latter case, the self-assembling nature of the TTF unit also allows the possibility of increased dimensionality within the polymer through interchain contacts, giving rise to enhanced bulk electronic properties. Whilst several groups have reported the synthesis and properties of CPs covalently linked to TTF units through non-conjugated links, some of us have been investigating oligothiophenes and polythiophenes that possess TTFs fused to the backbone of the conjugated chain (see **1**–**4** for example).^9^–^13^ In these studies, we have found that the fulvalene unit can either dominate the electroactivity of the polymer or participate in a true hybrid redox state, depending on the nature of the polymer structure.

In parallel to the above, the functional compound pyrrolo-TTF **5** and its derivatives^14^, ^15^ have attracted considerable attention as components in cyclophanes and cage molecules,^16^ molecular machines^20^–^22^

**Figure 1:**
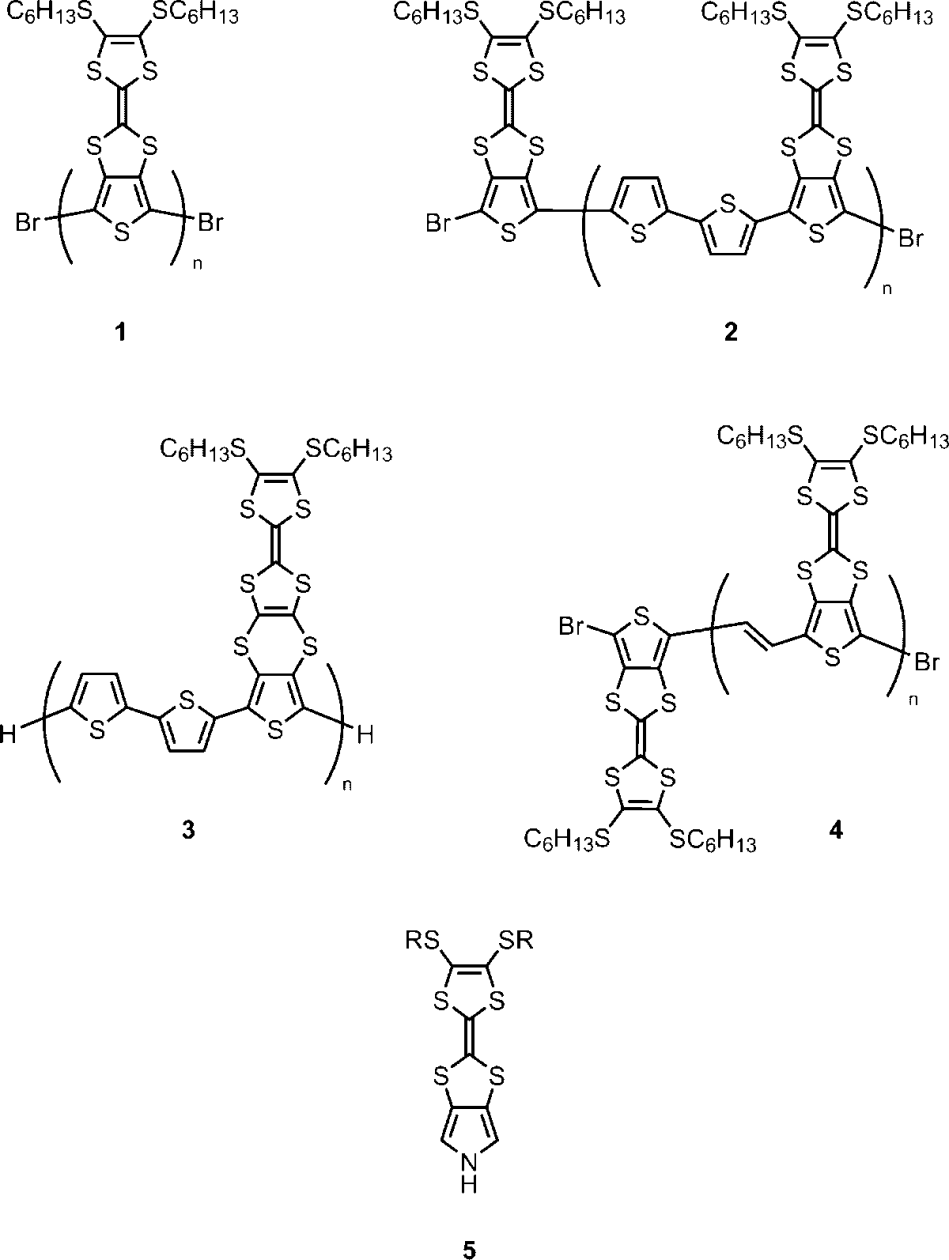
Structures of polymers 1–4 and compound 5.

Despite the strong interest in these materials, polymeric analogues have not yet been prepared and studied. Herein, we report the first synthesis of some copolymers containing the pyrrolo-TTF unit in the main chain and discuss the electronic and redox properties of the materials (Figure 1).

## Experimental Part

### Synthesis

**Scheme 1:**
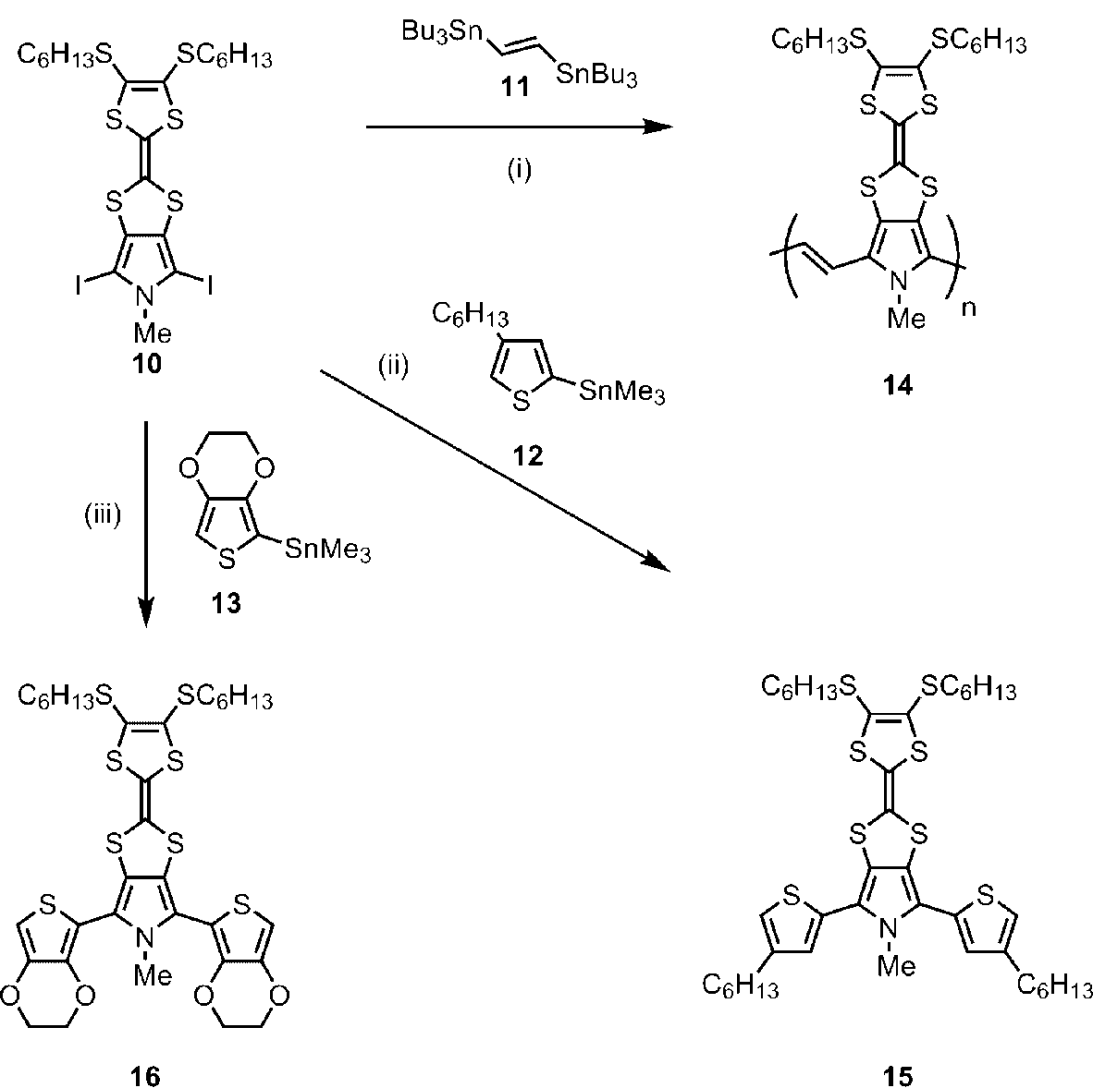
Reagents and conditions: (i) (EtO)_3_P, 130 °C, 4 h; (ii) (1) 3.0 equiv. LDA, THF, −78 °C, 30 min, (2) I_2_, −78 °C, 30 min, (3) rt, 30 min; (iii) 10 equiv. NaOMe, MeI, THF, rt, 30 min.

**Scheme 2:**
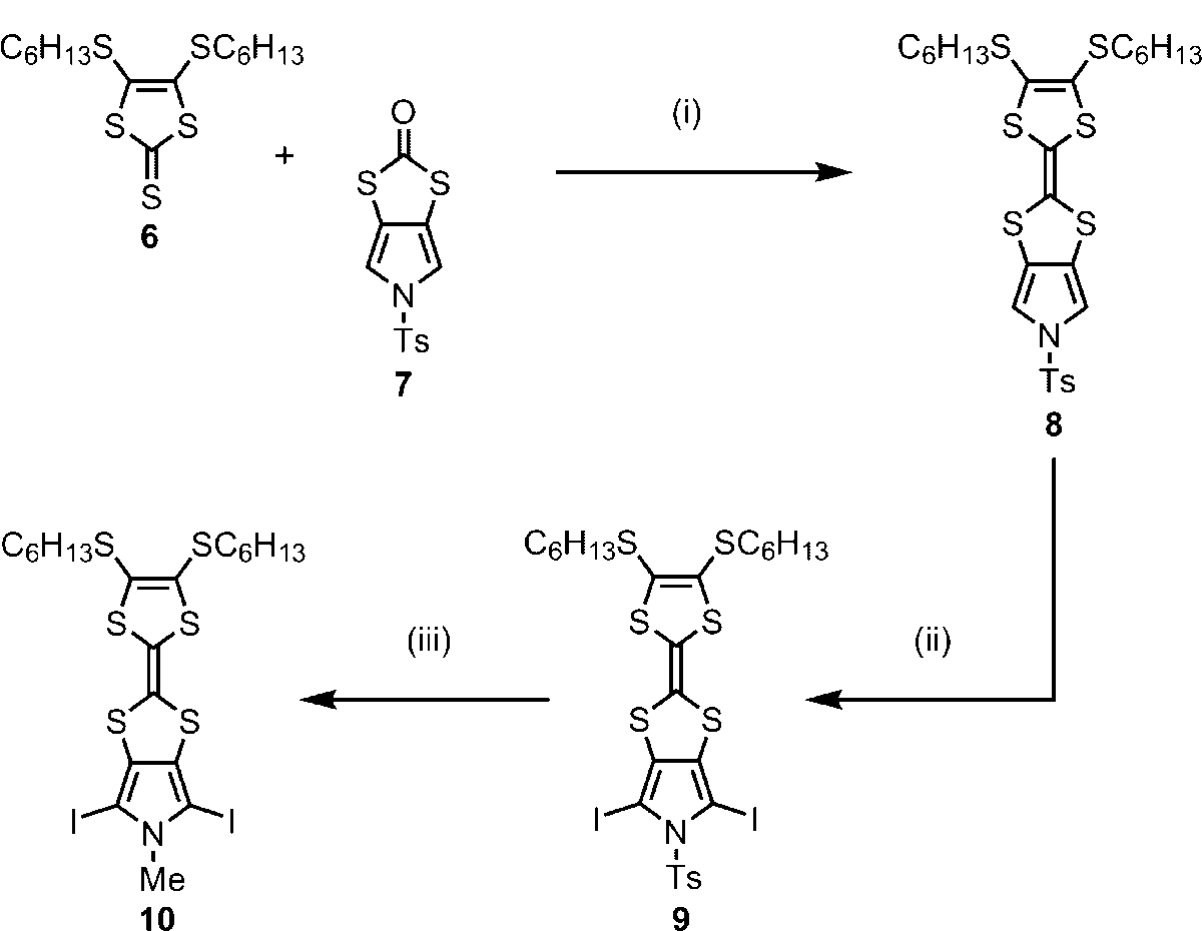
Reagents and conditions: (i) toluene, Pd(PPh_3_)_4_, reflux, 23 h; (ii) 2-trimethylstannyl-4-hexylthiophene, Pd(PPh_3_)_4_, DMF, µW, 160 °C, 1 h; (iii) 2-trimethylstannyl-3,4-ethylenedioxythiophene, Pd(PPh_3_)_4_, DMF, µW, 120 °C, 1 h.

The methodology used to prepare the new materials is depicted in Scheme 1 and 2. The key precursor 4,6-diiodo-5-methyl-2-[4,5-bis(hexylthio)-1,3-dithiole-2-ylidene-1,3-dithiolo][4,5-*c*]pyrrole (**10**) was synthesised as shown in Scheme 1. Cross-coupling of 5-tosyl-1,3-dithiolo[4,5-*c*]pyrrole-2-one^23^ (**7**) with 1.5 equiv. of 4,5-bis(hexylthio)-1,3-dithiole-2-thione (**6**) in neat triethyl phosphite gave **8** in 88% yield after column chromatography. Treatment of **8** with with 3 equiv. of lithium diisopropylamide (LDA) and subsequent reaction with iodine afforded the diiodo derivative **9** in 52% yield. Subsequently, in a one-pot reaction, the tosyl group in **9** was removed using sodium methoxide and the resulting pyrrole nitrogen was methylated using iodomethane, providing the key precursor **10** in 83% yield.^24^

Coupling (Scheme 2) of compound **10** with 1,2-bis(tributylstannyl)ethylene^25^ (**11**) using Pd(PPh_3_)_4_ as the catalyst and toluene as the solvent gave a dark purple mixture. The reaction mixture was poured into methanol and the resulting precipitate was subjected to Soxhlet extraction with methanol and acetone to remove impurities. Subsequent Soxhlet extraction with dichloromethane gave the target polymer as fraction **14a**, whilst a final extraction with chloroform gave **14b**. The yields for the two fractions were 142 mg (49%) and 7 mg (2%), respectively. Multi-angle light scattering GPC analysis of the main fraction (**14a**) in THF gave a molar mass of 317 500 g · mol^−1^, and the polydispersity was very narrow 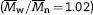.

Stille coupling methodology under microwave conditions was employed for the syntheses (Scheme 2) of compounds **15** and **16**. Thus, the reactions of 2-trimethylstannyl-4-hexylthiophene^26^ (**12**) and 2-trimethylstannyl-3,4-ethylenedioxythiophene^27^ (**13**) with the diiodo derivative **10** gave the corresponding products **15** and **16** in 69 and 46% yields, respectively.

### Electrochemistry

The redox behaviour of the monomers and polymers was assessed using cyclic voltammetry (see Table 1 for data, and Supporting Information for voltammograms). The triaryl monomers **14** and **16** display the usual two-step, reversible oxidation process to the TTF dication unit at similar potentials. A third, irreversible peak is observed at ca. +0.9 V, which is attributed to the oxidation of the bis(thienyl)pyrrole units. The irreversible nature of this redox event signifies the reactivity of the radical cation formed within the triaryl unit. Thus, repetitive cycling over a potential range covering the third oxidation waves of **14** and **16** resulted in the deposition of the corresponding polymers on the working electrode. In the case of poly(**16**), a new reversible peak emerges at +0.70 V, representing the electroactivity of the new polymer (see Supporting Information). The generation of this wave is in contrast to the redox behaviour of the electrochemically grown TTF-terthiophene analogue **3**, for which the polymer and TTF second oxidation peaks coalesce.^10^

Cyclic voltammograms of both polymers were obtained from films in monomer-free acetonitrile solution containing only electrolyte (0.1 m TBAPF_6_). Whilst poly(**16**) forms stable films, poly(**15**) dissolves readily during electrochemical experiments. In monomer-free solution, the second TTF oxidation of poly(**16**) and the oxidation of the conjugated chain coalesce, to give a relatively sharp peak at +0.85 V (see Supporting Information). In poly(**15**), however, the oxidation of the polymer chain remains distinguishable and 0.46 V higher than the TTF second oxidation. For both polymers, it is noticeable that the redox potentials for the TTF units are significantly higher than those in the parent monomers. This can be attributed to the electron withdrawing effect of the conjugated chain and the same trend was observed in the comparison of polymers **2** and **3** and their monomers.^10^ In contrast to the TTF units in polythiophene analogues **2** and **3**, the fulvalenes in poly[bis(thienyl)pyrrole]s poly(**15**) and poly(**16**) are stronger electron donors, with a difference in redox potentials approximately 0.3 V lower for the pyrrolo-TTFs.

The cyclic voltammograms for the polymer fractions **14a** and **14b** are identical and, coincidentally, very similar to that of poly(**15**). Again, the reversibility for the third oxidation is poor, but its presence is unexpected given that the PTV analogue **4** only displays two redox waves. The TTF units in **14a** and **14b** are stronger electron donors than in **4**, with the first and second oxidation potentials 0.4–0.5 V lower in the pyrrolo-TTFs. The results obtained from cyclic voltammetry experiments on **14a** and **14b** indicate that, contrary to the PTV material, the CP chain is redox active alongside the TTF units. Reduction processes for the polymers could not be detected since the materials were lost from the electrodes at negative potentials. The instability of the polymers to n-doping is in contrast to the detectable reduction processes of polymers **1**–**4**.

### Electronic Absorption Spectroscopy

Data from the absorption spectra of the new materials are collated in Table 1. The bathochromic shift of the longest wavelength absorption peak observed between the monomer **16** and poly(**16**) is expected and due to the elongation of the conjugated chain. However, the magnitude of the shift is low (Δ*λ*_max_ = 53 nm) and indicates a twisted conformation between aromatic units. As a useful comparison, polymers **2** and **3** give bathochromic shifts of 83 and 142 nm, respectively, compared to their terthiophene precursors.^10^ The band gap of poly(**16**) equates to 2.4 eV and is significantly wider than known EDOT-pyrrole copolymers, although the latter have only been prepared electrochemically as block copolymers from solutions containing two different monomers.^28^ The value is, however, within the range of homopolymers based on 3,4-alkylenedioxypyrroles^29^ and 3,4-alkylenedithiopyrroles.^30^ Due to the high solubility of poly(**15**) during electrochemical experiments and the consequent loss of material from the working electrode, only the absorption spectrum of the doped state (as-made polymer) could be recorded (normally dedoping of the polymer is performed by repetitive cycling through an inert potential range, between the first oxidation and reduction waves). Absorption maxima for poly(**15**) were observed at 372, 734 and 926 nm (see Supporting Information). The high energy peak can be assigned to the TTF dication (TTF^2+^ itself gives a peak at 390 nm),^6^ whilst the lower energy bands could arise from interchain charge transfer between radical cations sited within partially oxidised TTFs and polarons within the conjugated chain.^10^, ^31^

**Table 1:** Electronic absorption and electrochemical data in dichloromethane solutions for 14a,b, 15 and 16. In the case of poly(16), experiments were carried out on thin films deposited on ITO glass and the CV experiment was conducted in acetonitrile. All redox potentials are referenced to the ferrocene/ferrocenium redox couple. ^i^ Irreversible wave.

	Absorption Spectroscopy			Cyclic voltammetry		
	*λ*_max_/nm	Longest wavelength band onset/nm	Band gap/eV	*E* 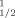 /V	*E* 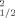 /V	*E* 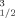 /V
**14a**	572, 328, 288	729	1.7	+0.42	+0.74	+1.12
**14b**	662, 604, 331, 282	736	1.7			
**15**	330	414	–	+0.08	+0.51	+0.96^i^
**Poly(15)**		See text		+0.41	+0.70	+1.16^i^
**16**	327, 276	409	–	+0.14	+0.55	+0.86^i^
**Poly(16)**	380	516	2.4	+0.40	+0.85	–

For polymer **14**, the longest wavelength absorption maximum is higher for the chloroform fraction (i.e., **14b**) and this is expected since chloroform normally isolates higher molecular fractions than dichloromethane. The band gap of 1.7 eV is wider than that of the thiophene analogue **4**, which is somewhat surprising given that the aromatic resonance energy for pyrrole is lower than thiophene and the expected quinoidal excited state should be easier to achieve for a pyrrole-containing polymer. However, the reduction in band gap for polythiophene analogues compared to polypyrroles is due to the greater difference in electron affinity, compared to the ionisation potential;^32^ therefore, the increased polarizability of the sulfur atom (vs. nitrogen) is responsible for the net effect in lowering the band gap. Oxidation of the polymer gave an absorption spectrum with major bands centred at 326, 551 and 867 nm, indicating that, in comparison with the spectro-electrochemical data from poly(**15**), the changes in the absorption spectrum are related to the polymer chain rather than the pyrrolo-TTF unit. Of particular note is the difference in the absorption spectrum of **14** compared with that of **4**, in which there is no doping of the polymer backbone.^10^

**Figure 2:**
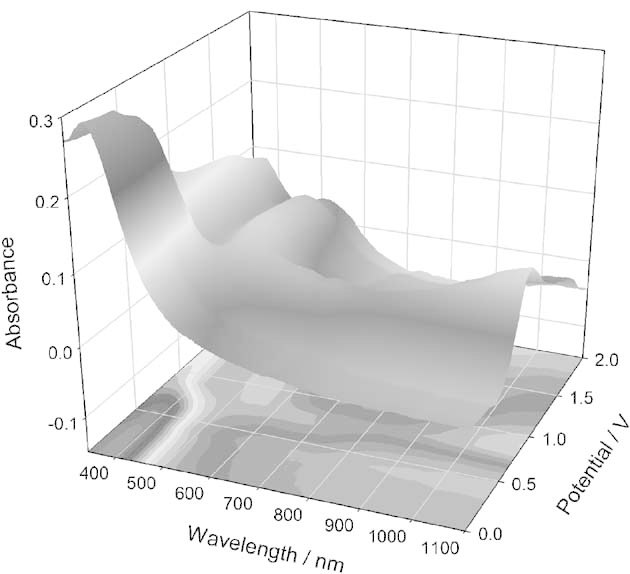
UV-vis-NIR spectroelectrochemical plot of poly(16) as a thin film on ITO glass.

The p-doping of poly(**16**) was investigated by UV–Vis–NIR spectro-electrochemistry and the spectra are summarised in a 3-D plot in Figure 2. The first oxidation takes place within the TTF unit and the corresponding electronic signatures of the TTF cation radicals begin to emerge as broad peaks at ca. 600 and 1 050 nm. The second oxidation peak of the polymer is quite sharp for a CP (see Supporting Information) and represents the simultaneous oxidation of the polymer chain and the second oxidation of the TTF unit. At a potential beyond the second oxidation wave, the TTF radical cation peaks disappear, a new band forms at around 640 nm and the main *π*–π* band diminishes significantly. In contrast to its closest thieno-TTF analogue, polymer **2**,^10^ poly(**16**) provides a well-defined series of absorption spectra upon doping. Within the predicted twisted geometry of the polymer chain, the bis-EDOT units will likely form planar subunits and, in this case, the oxidation of the main chain would be localised within these (EDOT)_2_ segments. Not only would this explain the well-defined absorption spectra of doped states, but it would also account for the sharp redox wave of the polymer at +0.85 V.

## Conclusion

In summary, a direct analogy has been made between polymers containing the pyrrolo-TTF unit and those possessing the related thieno-TTF. Within the series of polymers **1**–**4**, **14**, poly(**15**) and poly(**16**), the electrochemical dominance of the TTF units in the thieno-TTF analogues is lost in the pyrrolo-TTF polymers and the latter are wider band gap materials. The pyrrole rings lower the oxidation potentials of the TTF units compared to the thiophene analogues, but the N-containing polymers are less stable to n-doping.
